# Supplementary figures and images for: Habitat isolation interacts with top-down and bottom-up processes in a seagrass ecosystem
Source: PLoS One. 2023 Jul 26;18(7):e0289174. doi: 10.1371/journal.pone.0289174 (PMC10370773; doi:10.1371/journal.pone.0289174)

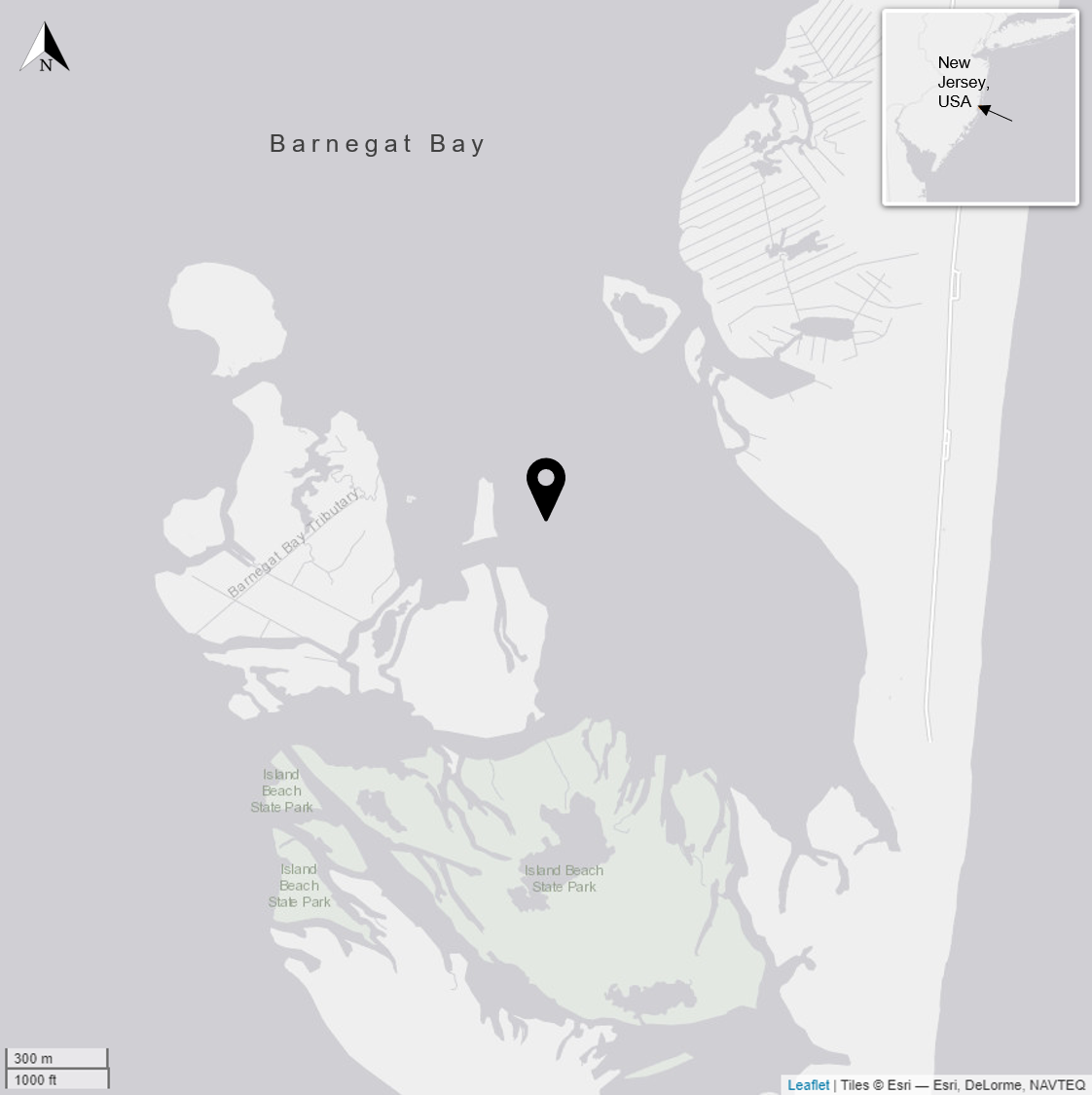

Supplement: S1 Fig — The study location (39°N 47’ 24.8064", 74°W 6’ 28.476") within Island Beach State Park, New Jersey, USA is indicated with a black pin. (TIF) [file pone.0289174.s001.tif]
