# Supplementary material for: Habitat isolation interacts with top-down and bottom-up processes in a seagrass ecosystem
Source: PLoS One. 2023 Jul 26;18(7):e0289174. doi: 10.1371/journal.pone.0289174 (PMC10370773; doi:10.1371/journal.pone.0289174)
Supplement: Attachment — Submitted filename: Response to Reviewers.docx [file pone.0289174.s002.docx]

May 31, 2023

Dear Dr. Bianchi,

Thank you very much for the opportunity to submit a revised manuscript for publication in PLOS ONE. We appreciate the comments we received during the review process, and we have aimed to address these points in our revised submission. Please see below for our point-by-point responses to reviewer comments in italics. We feel these revisions improved the clarity of the manuscript.

In addition, we wanted to clarify that all data used in this manuscript are publicly available in the Dryad data repository at the following DOI: 10.5061/dryad.3xsj3txm3. We appreciate you updating our Data Availability statement accordingly.

Thank you again for your consideration of our manuscript for publication in PLOS ONE.

Sincerely,

Elizabeth Carroll

*********************************

Dear Dr. Carroll,

Thank you for submitting your manuscript to PLOS ONE. After careful consideration, we feel that it has merit but does not fully meet PLOS ONE’s publication criteria as it currently stands. Therefore, we invite you to submit a revised version of the manuscript that addresses the points raised during the review process.

Both reviewers highlighted methodological issues (sampling design, use of transects, etc.) that must be fixed before publication. In addition, I noted that “ppt” has not been in use for salinity measures since decades: please check if you can adopt PSU, instead.

*We have adopted the use of PSU in the text. Given the temperature range for our site, no conversion was necessary.*

Please submit your revised manuscript by Jun 01 2023 11:59PM. If you will need more time than this to complete your revisions, please reply to this message or contact the journal office at [plosone@plos.org](mailto:plosone@plos.org). When you're ready to submit your revision, log on to <https://www.editorialmanager.com/pone/> and select the 'Submissions Needing Revision' folder to locate your manuscript file.

Please include the following items when submitting your revised manuscript:

- A rebuttal letter that responds to each point raised by the academic editor and reviewer(s). You should upload this letter as a separate file labeled 'Response to Reviewers'.
- A marked-up copy of your manuscript that highlights changes made to the original version. You should upload this as a separate file labeled 'Revised Manuscript with Track Changes'.
- An unmarked version of your revised paper without tracked changes. You should upload this as a separate file labeled 'Manuscript'.

If you would like to make changes to your financial disclosure, please include your updated statement in your cover letter. Guidelines for resubmitting your figure files are available below the reviewer comments at the end of this letter.

If applicable, we recommend that you deposit your laboratory protocols in [protocols.io](http://protocols.io/) to enhance the reproducibility of your results. Protocols.io assigns your protocol its own identifier (DOI) so that it can be cited independently in the future. For instructions see: <https://journals.plos.org/plosone/s/submission-guidelines#loc-laboratory-protocols>. Additionally, PLOS ONE offers an option for publishing peer-reviewed Lab Protocol articles, which describe protocols hosted on [protocols.io](http://protocols.io/). Read more information on sharing protocols at <https://plos.org/protocols?utm_medium=editorial-email&utm_source=authorletters&utm_campaign=protocols>.

We look forward to receiving your revised manuscript.

Kind regards,

Carlo Nike Bianchi
Academic Editor
PLOS ONE

Journal Requirements:

When submitting your revision, we need you to address these additional requirements.

1. Please ensure that your manuscript meets PLOS ONE's style requirements, including those for file naming. The PLOS ONE style templates can be found at

<https://journals.plos.org/plosone/s/file?id=wjVg/PLOSOne_formatting_sample_main_body.pdf> and

<https://journals.plos.org/plosone/s/file?id=ba62/PLOSOne_formatting_sample_title_authors_affiliations.pdf>

*Our manuscript now adheres to these requirements.*

2. Please provide additional details regarding participant consent. In the ethics statement in the Methods and online submission information, please ensure that you have specified (1) whether consent was informed and (2) what type you obtained (for instance, written or verbal, and if verbal, how it was documented and witnessed). If your study included minors, state whether you obtained consent from parents or guardians. If the need for consent was waived by the ethics committee, please include this information.

If you are reporting a retrospective study of medical records or archived samples, please ensure that you have discussed whether all data were fully anonymized before you accessed them and/or whether the IRB or ethics committee waived the requirement for informed consent. If patients provided informed written consent to have data from their medical records used in research, please include this information.

*No human subjects were used in this research.*

3. In your Data Availability statement, you have not specified where the minimal data set underlying the results described in your manuscript can be found. PLOS defines a study's minimal data set as the underlying data used to reach the conclusions drawn in the manuscript and any additional data required to replicate the reported study findings in their entirety. All PLOS journals require that the minimal data set be made fully available. For more information about our data policy, please see <http://journals.plos.org/plosone/s/data-availability>.

Upon re-submitting your revised manuscript, please upload your study’s minimal underlying data set as either Supporting Information files or to a stable, public repository and include the relevant URLs, DOIs, or accession numbers within your revised cover letter. For a list of acceptable repositories, please see <http://journals.plos.org/plosone/s/data-availability#loc-recommended-repositories>. Any potentially identifying patient information must be fully anonymized.

Important: If there are ethical or legal restrictions to sharing your data publicly, please explain these restrictions in detail. Please see our guidelines for more information on what we consider unacceptable restrictions to publicly sharing data: <http://journals.plos.org/plosone/s/data-availability#loc-unacceptable-data-access-restrictions>. Note that it is not acceptable for the authors to be the sole named individuals responsible for ensuring data access.

We will update your Data Availability statement to reflect the information you provide in your cover letter.

*All data used in this research are publicly available, and we have included this information in our cover letter above.*

[Note: HTML markup is below. Please do not edit.]

Reviewers' comments:

Reviewer's Responses to Questions

**Comments to the Author**

1. Is the manuscript technically sound, and do the data support the conclusions?

The manuscript must describe a technically sound piece of scientific research with data that supports the conclusions. Experiments must have been conducted rigorously, with appropriate controls, replication, and sample sizes. The conclusions must be drawn appropriately based on the data presented.

Reviewer #1: Yes

Reviewer #2: Yes

2. Has the statistical analysis been performed appropriately and rigorously?

Reviewer #1: Yes

Reviewer #2: Yes

3. Have the authors made all data underlying the findings in their manuscript fully available?

The [PLOS Data policy](http://www.plosone.org/static/policies.action#sharing) requires authors to make all data underlying the findings described in their manuscript fully available without restriction, with rare exception (please refer to the Data Availability Statement in the manuscript PDF file). The data should be provided as part of the manuscript or its supporting information, or deposited to a public repository. For example, in addition to summary statistics, the data points behind means, medians and variance measures should be available. If there are restrictions on publicly sharing data—e.g. participant privacy or use of data from a third party—those must be specified.

Reviewer #1: Yes

Reviewer #2: Yes

4. Is the manuscript presented in an intelligible fashion and written in standard English?

PLOS ONE does not copyedit accepted manuscripts, so the language in submitted articles must be clear, correct, and unambiguous. Any typographical or grammatical errors should be corrected at revision, so please note any specific errors here.

Reviewer #1: Yes

Reviewer #2: Yes

5. Review Comments to the Author

Please use the space provided to explain your answers to the questions above. You may also include additional comments for the author, including concerns about dual publication, research ethics, or publication ethics. (Please upload your review as an attachment if it exceeds 20,000 characters)

Reviewer #1: The manuscript “Habitat isolation interacts with top-down and bottom-up processes in a seagrass ecosystem” showcase an interesting and current topic worthy to be object of detailed studies. Hypotheses are well argued in the introduction; however, I believe the authors could provide a clearer description of the sampling design used and add a cautionary paragraph in the discussion to critically consider weakness and results of the study.
Below you will find more detailed comments and suggestions.

*Thank you for these comments. Please see our responses below.*

Lines 77-81: I suggest to add more recent and updated references to support these statements.

*Thank you for your suggestion. We’ve updated the manuscript to include additional recent papers which quantify global losses of key marine foundation species such as kelp, coral, mangroves, and seagrass.*

Lines 113-123: It is not clear where are located the three field sites in which the study has been carried out. Are all the three sites included in the Island Beach State Park meadow? Maybe a map and a more detailed description of the study area could help.

*Thank you for identifying this point of confusion. We completed three field studies at the same field site in Island Beach State Park. We updated the manuscript to include more detailed information about this study location, and we now include a map of our study site as a supplemental figure.*

Lines 127-129: were the five transects repeated in each field sites (x 3)? The sampling has been carried out in August, are epibionts affected by the seasonality?

*There is only one field site which consists of a large continuous meadow of seagrass surrounded by patches of seagrass. Transects ran from the meadow into the patches. We have revised the text in this section to improve clarity. Epibiont recruitment can be influenced by seasonality. Recruitment occurs from May to October in this region, with peak recruitment occurring during the summer months (June-August). Therefore, the epibiont composition in August reflects the period of peak recruitment and productivity. We modified the manuscript text in this section to clarify this point.*

Lines 129-132: The sampling design is not clear, 10 blades at the beginning of each transect so totally 50 blades? X 3 site (150)? They represent the sampling effort for the “continuous meadow”, and what about the “isolated” and “very isolated”? 18 patches have been found, totally? How many isolated and very isolated? Please clarify the sampling design and the sampling effort.

*We’ve revised this section to provide more detail on the sampling effort for the natural seagrass analysis.*

Lines 148-151: If it is available, I suggest to add a picture of the placed ASUs.

*Thank you for this suggestion, but unfortunately, we do not have a publication-quality image to accompany our paper. However, ASUs are a commonly used approach in seagrass studies, and there are other images available online that demonstrate this approach.*

Lines: 153-154: How many ASUs at each isolation level?

*We’ve clarified in the manuscript that twenty ASUs were placed at each of three isolation levels for a total of 60 ASUs.*

*We have also included S2 Fig as a supplementary file that shows the spatial arrangement of ASUs in our study.*

Lines 234-235: I suggest to not speak of the results in the caption of the figure (applies to all figure captions).

*Thank you for this suggestion, however, we feel that highlighting the main result for each figure assists readers and clarifies our primary findings. Therefore, our preference is to retain this approach in the manuscript.*

Lines 256-259: same comment (see above). As you always consider three levels of isolation, I suggest to uniform them in all the graphs (i.e., meadow, low isolation, high isolation), then you can specify in the caption to which distance they refer to.

*Thank you for pointing out this inconsistency in the x-axis of Figure 1. We’ve updated all x-axes to be consistent in the graphs.*

Lines 320-321: add references.

*This sentence reflects the main finding of our study, as justified by the subsequent sentences in that initial paragraph of our discussion.*

Reviewer #2: The paper by Carroll and Freestone aim at evaluating how habitat isolation modifies community structure and mediates top-down and bottom-up control of primary producers in a marine seagrass system. The topic is interesting and the study is rightly conducted, as data are to be published, although some changes are needed to improve the paper. A list of comment/suggestions is reported below.

Line 95: “Our aim was to use seagrass meadows, a system of high conservation importance, to explore two questions”

*Thank you, we’ve adopted this text.*

Line 121: “temperature from 21 to 25.3°C, and salinity from 20 to 25 ppt” temporal reference for these values should be given

*We’ve added a statement about the time period of the study.*

Lines 124-142, Habitat isolation in natural seagrass: the sampling method should be better clarified.
How many replicates for each condition? Five? What is the logic to use transects if these latter are not considered in the analyses? Why do not consider three habitats and collect five replicates for each habitats? Transects should be justified or removed

*Transects were used as a method of surveying the area for frequency and distribution of patches around the continuous meadow as well as determining appropriate distances for the artificial seagrass experiment. We’ve added a statement to the methods about the use of transects. We’ve reanalyzed our data to include transect as a random effect in our models. However, this factor was not significant (P>0.8) and was therefore removed from our final models. We now detail this in the manuscript.*

Line 143, Habitat isolation and consumer-resource experiment: the use of artificial blades could potentially modify the recruitment pattern compared to natural blades. This aspect should be at least considered in the discussion.

*We’ve added text regarding the use of ASUs in the discussion (now Lines 413-419 in the track changes document).*

Lines 171: The analyses of nutrient levels in water column in ASU and control plots are needed to evaluate the nutrient increase; if the authors have no data, they should at least provide bibliographic references about the use of the same method to quantify the nutrient enhancing in seagrass systems (e.g Journal of Sea Research 63 (2010) 173-179; Scientific Reports (2017) 7, 13732)

*We’ve incorporated the suggested Scientific Reports reference and another to the manuscript which is now Line 186.*

6. PLOS authors have the option to publish the peer review history of their article ([what does this mean?](https://journals.plos.org/plosone/s/editorial-and-peer-review-process#loc-peer-review-history)). If published, this will include your full peer review and any attached files.


If you choose “no”, your identity will remain anonymous but your review may still be made public.


**Do you want your identity to be public for this peer review?** For information about this choice, including consent withdrawal, please see our [Privacy Policy](https://www.plos.org/privacy-policy).

Reviewer #1: No

Reviewer #2: No
