# Supplementary material for: Habitat isolation interacts with top-down and bottom-up processes in a seagrass ecosystem
Source: PLoS One. 2023 Jul 26;18(7):e0289174. doi: 10.1371/journal.pone.0289174 (PMC10370773; doi:10.1371/journal.pone.0289174)
Supplement: Attachment — Submitted filename: Comments reviewer_1.docx [file pone.0289174.s003.docx]

Comments

I have read and appreciated revisions the authors have made to the text but I still find some points of confusion that I suggest to clarify for the readers. It is now clear to me that transects were only used as a mean of identifying patches around the main body of the meadow and to define their distance from it: I believe that the supplementary figure 2 well explains the concept idea behind the study and it should be added to the main text. Eventually, patches could be defined in terms of size (what is the minimum size to define a patch or the average size order)

Talking about three field studies at the beginning of methods can be confusing, e.g. initially I thought the authors meant 3 replicates. I suggest specifying that these are three separate studies to investigate 1) Habitat isolation in natural seagrass; 2) Habitat isolation vs. consumer-resource 3) Recruitment.
